# Enhanced Anti-*Mycobacterium tuberculosis* Immunity over Time with Combined Drug and Immunotherapy Treatment

**DOI:** 10.3390/vaccines6020030

**Published:** 2018-05-24

**Authors:** Sasha E. Larsen, Susan L. Baldwin, Mark T. Orr, Valerie A. Reese, Tiffany Pecor, Brian Granger, Natasha Dubois Cauwelaert, Brendan K. Podell, Rhea N. Coler

**Affiliations:** 1 Infectious Disease Research Institute, Seattle, WA 98102, USA; Sasha.Larsen@idri.org (S.E.L.); Susan.Baldwin@idri.org (S.L.B.); Mark.Orr@idri.org (M.T.O.); Valerie.Reese@idri.org (V.A.R.); Tiffany.Pecor@idri.org (T.P.); Brian.Granger@idri.org (B.G.); Natasha.Dubois@gmail.com (N.D.C.); 2 Department of Global Health, University of Washington, Seattle, WA 98195, USA; 3 Department of Microbiology, Immunology and Pathology, Colorado State University, Fort Collins, CO 80523, USA; Brendan.Podell@ColoState.edu; 4 PAI Life Sciences Inc., Seattle, WA 98102, USA

**Keywords:** therapeutic vaccine, *Mycobacterium tuberculosis*, tuberculosis, immunotherapy, adjuvant, GLA-SE, ID93

## Abstract

It is estimated that one third of the world’s population is infected with *Mycobacterium tuberculosis* (*Mtb*). This astounding statistic, in combination with costly and lengthy treatment regimens make the development of therapeutic vaccines paramount for controlling the global burden of tuberculosis. Unlike prophylactic vaccination, therapeutic immunization relies on the natural pulmonary infection with *Mtb* as the mucosal prime that directs boost responses back to the lung. The purpose of this work was to determine the protection and safety profile over time following therapeutic administration of our lead *Mtb* vaccine candidate, ID93 with a synthetic TLR4 agonist (glucopyranosyl lipid adjuvant in a stable emulsion (GLA-SE)), in combination with rifampicin, isoniazid, and pyrazinamide (RHZ) drug treatment. We assessed the host inflammatory immune responses and lung pathology 7–22 weeks post infection, and determined the therapeutic efficacy of combined treatment by enumeration of the bacterial load and survival in the SWR/J mouse model. We show that drug treatment alone, or with immunotherapy, tempered the inflammatory responses measured in brochoalveolar lavage fluid and plasma compared to untreated cohorts. RHZ combined with therapeutic immunizations significantly enhanced TH1-type cytokine responses in the lung over time, corresponding to decreased pulmonary pathology evidenced by a significant decrease in the percentage of lung lesions and destructive lung inflammation. These data suggest that bacterial burden assessment alone may miss important correlates of lung architecture that directly contribute to therapeutic vaccine efficacy in the preclinical mouse model. We also confirmed our previous finding that in combination with antibiotics therapeutic immunizations provide an additive survival advantage. Moreover, therapeutic immunizations with ID93/GLA-SE induced differential T cell immune responses over the course of infection that correlated with periods of enhanced bacterial control over that of drug treatment alone. Here we advance the immunotherapy model and investigate reliable correlates of protection and *Mtb* control.

## 1. Introduction

Tuberculosis (TB), caused by the intracellular bacterium *Mycobacterium tuberculosis* (*Mtb*), was the leading cause of death from a single pathogen for the third year in a row, killing 1.3 million HIV-negative people in 2016 [1]. Up to 16% of people infected with TB die from the disease each year and the WHO reported 6.3 million new TB cases in 2016, up from 6.1 million in 2015. Increasingly worrisome is its ability to develop extensive drug resistance [1]. At present there is a desperate need for successful and durable vaccines against *Mtb*. The current TB vaccine for much of the world is Bacillus Calmette-Guérin (BCG). While BCG provides limited protection through adolescence from disseminated disease, it fails to protect individuals from adult pulmonary disease, which is the most transmissible form of the disease [2]. This puts the burden of disease control on complex 6+ month multidrug treatment regimens [3]. Unfortunately, extensive schedules of poorly-tolerated drugs can limit patient compliance and promote the development of drug resistance, requiring even longer drug treatment regimens, and perpetuate infectious distribution. Novel treatments and preventative vaccines effective against drug-sensitive (DS) and drug-resistant (DR) strains would significantly impact the global burden of this disease.

To date, there have been limited anti-TB vaccines that have entered phase 2b or phase 3 clinical trials, and those that have made it through rigorous clinical trial hurdles have failed at later stages [4,5,6]. To compound the issues faced by the TB research community, durable immune correlates of protection remain elusive, making advancement of novel vaccine candidates difficult. Traditionally, strong T helper 1 (TH1) phenotypes have been used as a standard measure of *Mtb*-specific responses post vaccination or infection. However, these immune parameters are quite complex and require more scrutiny than simply assessing whether these populations are present or absent. Recent evidence suggests anti-mycobacterial TH1 responses are most beneficial in moderation [7], are not singularly responsible for protection afforded by vaccination [8], and are not the only population that can control *Mtb*. In this publication we specifically assess the kinetics of anti-mycobacterial TH1 responses to determine if they contribute during specific intervals of bacterial control. Additionally, memory and regulatory immune subsets have been critically underappreciated for their contributions to *Mtb* control [9]. Most recently, less differentiated subsets of antigen-specific CD4^+^ T cells have demonstrated rapid recall responses and protection in mice for up to 40 weeks post challenge in the context of ESAT-6 vaccination [10], but have yet to be validated in the non-human primate model for translational protective efficacy. Methods to generate less differentiated, protective cells from memory subsets and protective immunity against *Mtb* remains an area of active investigation. To date, it is not clear if these subsets contribute to controlling the bacterial burden at different times during infection.

Our novel first-generation vaccine candidate, ID93, a fusion of Rv2608, Rv3619, Rv3620, and Rv1813 *Mtb* antigens, combined with synthetic toll-like receptor 4 (TLR4) agonist glucopyranosyl lipid adjuvant (GLA) in a stable nano-emulsion (SE), is an effective prophylactic TB vaccine which has been tested in several preclinical animal models, demonstrating protection against lab-adapted and virulent clinical isolates of *Mtb*, as well as DS and DR *Mtb* strains, and is currently in phase 2 clinical trials [11,12,13,14,15,16,17,18,19,20] (trial identifiers: NCT01599897, NCT02508376). Most recently ID93/GLA-SE demonstrated safety and immunogenicity in BCG-vaccinated healthy adults with both positive and negative QuantiFERON status (defining active or latent *Mtb* infection) in an *Mtb* endemic region of South Africa [21] (trial identifier: NCT01927159), inferring potential as a therapeutic candidate. We have previously demonstrated enhanced survival after therapeutic immunization with ID93/GLA-SE in the preclinical mouse model and non-human primates in combination with dual drug (rifampicin and isoniazid (RH)) treatment [17]. The specific purpose of this investigation was to build upon these results and determine the kinetics of protective efficacy with therapeutic ID93/GLA-SE in combination with front-line antibiotics (rifampicin, isoniazid and pyrazinamide (RHZ)), and how this treatment regimen affects bacterial burden, influences host inflammatory immune responses, and impacts pulmonary immunopathology over time. Here, we have confirmed enhanced survival against *Mtb* with a combined drug and immunotherapy approach. We also show decreased non-specific inflammatory responses in the lung and circulation and increased vaccine-specific cellular immunity that correspond to superior bacterial control and reduced lung immunopathology at different timepoints following this treatment regimen. Importantly, we demonstrate with a kinetic investigation that TH1 responses play a critical role in early/intermediate control of bacterial burden and that therapeutic vaccination with ID93/GLA-SE may enhance survival by maintaining lung architecture versus reducing bacterial loads over time.

## 2. Materials and Methods

*Preclinical animal model.* Female SWR/J mice 4–6 weeks of age were purchased from Jackson Laboratory (Sacramento, CA, USA). Mice were housed under pathogen-free conditions at the Infectious Disease Research Institute (IDRI) biosafety level 3 animal facility and were handled in accordance with the specific guidelines of IDRIs Institutional Animal Care and Use Committee. Mice were infected with a low dose (50–100 bacteria) aerosol (LDA) of *Mycobacterium tuberculosis* H37Rv using a University of Wisconsin-Madison aerosol chamber. Twenty-four hours post challenge the lungs of three mice were homogenized and plated on Middlebrook 7H10 agar (Molecular Toxicology, Boone, NC, USA) to confirm delivery of 50–100 CFU per mouse. Four weeks post challenge specific cohorts of animals began treatment with chemotherapy (rifampicin 100 mg/L (Chem-Impex, Wood Dale, IL, USA), isoniazid 250 mg/L, and pyrazinamide 150 mg/L (ACROS Organics/Thermo Fischer Scientific, Waltham, MA, USA)) for eight weeks total, delivered ad libitum in the drinking water, while some mice remained untreated.

*Vaccines and adjuvants*. Cohorts of mice were immunized intramuscularly (i.m.) three times three weeks apart beginning 6 weeks post challenge (two weeks into drug treatment). Mice received either saline alone, or vaccinations containing 0.5 µg/dose of ID93 recombinant fusion protein combined with 5.0 µg/dose TLR4 stimulant glucopyranosyl lipid adjuvant (GLA), formulated in a squalene emulsion (SE), as previously published [13,15].

*Flow cytometry*. Intracellular flow cytometry was performed on lung homogenates 7, 10, 12, 16, 22, and 23 weeks post *Mtb* infection. Samples were incubated in red blood cell lysis buffer (eBioscience/Thermo Fischer Scientific, Waltham, MA, USA), washed and resuspended in RPMI 1640 (Life Technologies, Carlsbad, CA, USA) + 10% fetal bovine serum (FBS) (BioWhittaker, Inc./Lonza Radnor, PA, USA), and subsequently aliquotted in 96-well round-bottom plates. Cells were then stimulated with media alone, 10 µg/mL of recombinant ID93, or 1 µg/mL phorbol myristate acetate (PMA) (Calbiochem) + 1 µg/mL ionomycin (Sigma-Aldrich, St. Louis, MO, USA) and incubated at 37 °C. After one to two hours 1 µg/µL of GolgiPlug (BD Biosciences) was added and samples were incubated at 37 °C for an additional 8 h. Samples then remained at 4 °C until staining. Samples were first surface stained with flourochrome-conjugated antibodies against mouse CD4 (clone RM4-5, BioLegend, San Diego, CA, USA), CD8 (clone 53-6.7, BioLegend) and 1 µg/mL of Fc receptor block anti-CD16/CD32 (clone 93, eBioscience), with Live/Dead stain (Invitrogen, /Thermo Fischer Scientific, Waltham, MA, USA) in PBS with 1% bovine serum albumin (BSA) for 10–15 min at room temperature (RT). Cells were then washed and fixed using BD Biosciences Fix/Perm reagent for 20 min at RT. Subsequently, samples were washed with BD Perm/Wash followed by intracellular staining in Perm/Wash reagent with anti-mouse GMCSF (clone MP1-22E9, BioLegend), IFN-γ (clone XMG1.2, Invitrogen), IL-2 (clone JES6-5H4, eBioscience), IL-17A (clone Tc11-1BH10.1, BioLegend), TNF-α (clone MP6-XT22, eBioscience), IL-5 (clone TRFK5, eBioscience), and CD154 (clone MR7, eBioscience) for 10 min at RT. For assessing regulatory T cells (Treg), cells were similarly surface stained as described above with anti-mouse CD4, CD8, CD19 (clone 6D5, BioLegend), CD25 (clone PC61.5, eBioscience) and Live/Dead stain for 10 min at RT. Samples were washed and incubated in FoxP3 Fix/Perm for one hour at RT. Samples were washed and then stained overnight with anti-mouse FoxP3 (clone FJK-16S, eBioscience). Before removing samples from the BSL3, samples were incubated in 4% paraformaldehyde for 20 min. All antibodies were used at 10 µL/mL. After wash and resuspension in PBS + 1% BSA, samples were acquired on a BD Bioscience LSRFortessa flow cytometer (BD Bioscience, San Jose, CA, USA).

*Bacterial burden/CFU*. Four to 23 weeks post infection with *Mtb*, 4–7 mice per group were euthanized with CO_2_. Lung and spleen tissue from infected animals was isolated and homogenized in 5 mL of either RPMI + FBS (lung) or PBS + Tween-80 (Sigma-Aldrich, St. Louis, MO, USA) CFU buffer (spleen) using an Omni tissue homogenizer (Omni International, Kennesaw, GA, USA). Serial dilutions of homogenate were made in CFU buffer and aliquots were plated on Middlebrook 7H10 agar plates and subsequently incubated at 37 °C and 5% CO_2_ for 2–3 weeks before colonies were counted. Bacterial burden, as CFU/mL, was calculated per organ and is presented here as Log_10_ values. Reduction in the bacterial burden was calculated as the difference in mean Log10 values between groups assessed.

*Histology.* Lung accessory lobes were collected at different time points after *Mtb* challenge and were immediately perfused and subsequently stored in 10% normal buffered formalin. Fixed lungs were embedded in paraffin, cut and slides were generated with either hematoxylin and eosin (H and E) staining or Fite’s acid-fast staining at the Benaroya Research Institute Histology Core facility (Seattle, WA, USA). Stained slides were blinded and analyzed by a veterinary pathologist (Dr. Brendan Podell, Colorado State University). Quantification of pulmonary lesion burden was performed using the Stereo Invesitgator area morphometry function, as previously described [13,14]. Seventeen to 26 counting frames were sampled for each mouse lung section and were assigned randomly by the software depending on tissue size, and a counting frame of 2.5 × 10^5^ μm^2^ with a grid spacing of 50 μm used to define the areas of interest. Data were expressed as a percentage ratio of lesion to total tissue area. Mice were provided a semiquantitative pathology score based on three major categories, including the extent and frequency of granulomas, the cellular composition of granulomas, and the degree of destructive inflammatory pathology. For the extent and frequency of granuloma lesions, mice were assigned a score of 1–4 based on an overall burden of <10%, 10–25%, 25–50%, or 50–75%, respectively. In evaluating cellular composition of lesions, mice were assigned a score of 1–4 based on lymphocyte composition of >50%, 25–50%, <25%, or essentially absent, respectively, with higher scores applied for lesions dominated by macrophages and having increased frequency of neutrophils. Destructive inflammatory pathology was scored on a scale of 1–4, based on the presence of well-delineated granulomas, significant extension of inflammatory cells into surrounding alveolar spaces or airway walls, or extensive inflammation with coalescence of lesions as well as individual cell or foci of necrosis, and accumulation of proteinaceous fluid or fibrin. Collectively, a scale of severity is based on a minimum score of 3 and a maximum score of 12 for each animal, with 12 being the most severe pathology. Data were expressed as the mean score for each treatment group ± SD.

*Luminex.* A mix and match Procarta 26-plex mouse analyte kit (Thermo Fisher Scientific, Waltham, MA, USA) was used according to manufactures instructions for the detection of the following analytes in plasma and bronchoalveolar lavage fluid (BALF) samples collected at various time points post *Mtb* challenge: CXCL5 (ENA-78), GM-CSF, IFN-α, IFN-γ, IL-1α, IL-1β, IL-2, IL-2RA, IL-4, IL-5, IL-6, IL-7, IL-7RA, IL-9, IL-10, IL-12p70, IL-13, IL-15/IL-15R, IL-17a, IL-21, IL-23, IP-10, Leptin, MCP-1, MIP1-β, and TNF-α. Assays were read on the Luminex 200 and Luminex SD system and analysis was done according to protocol with respect to standards.

*CRP ELISA.* Mouse C-reactive protein levels were detected in serum samples from mice in cohorts previously described using an R&D systems Quantikine ELISA according to kit protocol. Amounts were determined as ng/mL in relationship to kit provided standards.

*Statistics.* Intracellular cytokines induced after T cell restimulation, cytokines present in BALF and plasma samples, bacterial burden and pathology assessments over time were compared between treatment groups using two-way ANOVA with Sidak’s multiple comparison test. Bacterial burden at a single time point was assessed using one-way ANOVA with Sidak’s multiple comparison test compared to untreated samples. Flow cytometry data was assessed using FlowJo v10 (BD) and SPICE (NIH) using the Wilcoxon signed rank test as compared to untreated groups. Survival curves were evaluated using a Log rank (Mantel-Cox) test. Statistical analyses, aside from flow cytometry, were performed using GraphPad Prism 7 software. *p* values < 0.05 were considered significant and labeled accordingly in the figures.

## 3. Results

### 3.1. Strong CD4^+^ TH1 T Cell Inflammatory Responses Elicited over Time in Groups Receiving RHZ and Immunotherapy

**Figure 1:**
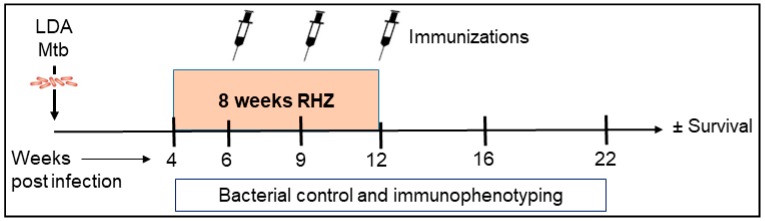
Preclinical mouse model of therapeutic vaccination against *Mtb*. SWR/J mice were challenged with a low-dose aerosol (LDA) of H37Rv *Mtb*. Four weeks post exposure, cohorts of mice were given RHZ antibiotic treatment for a total of eight weeks. In addition to RHZ treatment, a subset of mice also received ID93/GLA-SE immunizations by the i.m. route (three times at three week intervals) beginning six weeks post infection. From week 4–22 post challenge with *Mtb*, cohorts of 4–7 mice were assessed for bacterial burden, safety, and immune profiles. Survival was evaluated in 10 mice per group for 280 days.

**Figure 2:**
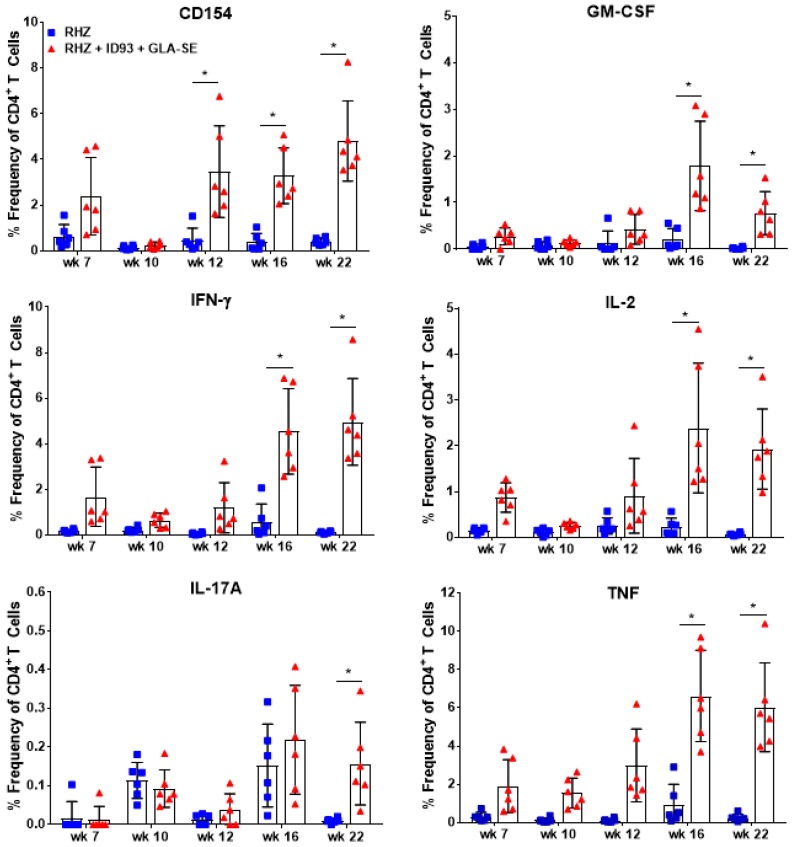
Cytokine production from ID93 antigen-specific CD4^+^ T cells over time. Percent frequency of CD4^+^ T cells expressing CD154, GM-CSF, IFN-γ, IL-2, IL-17A, and TNF cytokines after restimulation with ID93 antigen in samples collected in RHZ only (blue, squares) treated and RHZ+ID93/GLA-SE (red, triangles) treated cohorts over time. Significant differences between cohorts was determined by two-way ANOVA with Sidak’s multiple comparison test. The asterisk denotes significance where *p* < 0.05. Six mice/group/timepoint were included and data are representative of two independent experiments.

In order to assess the efficacy and immunological consequences of therapeutic vaccination with ID93/GLA-SE during antibiotic treatment (RHZ) we utilized the SWR/J mouse for their particular susceptibility to *Mtb* H37Rv infection [22,23,24]. We expected significant changes over time in the host immune response post challenge with and without treatment (Figure 1). To evaluate immunogenicity, cells were isolated from lung homogenate, stimulated ex vivo with vaccine antigen (ID93) and comparisons were made between RHZ only and RHZ+ID93/GLA-SE cohorts. The additive responses observed over time in comparison to RHZ alone are strongly inferred effects due to therapeutic immunization (Figure 2).

From 4–22 weeks post infection CD4^+^ T cells isolated from the lung were assessed for ex vivo production of TH1 cytokines by intracellular flow cytometry (Figure S1, Supplementary Materials). Despite beginning therapeutic immunizations at 6 weeks post infection, CD154^+^ activated, vaccine-specific CD4^+^ T cells from cohorts of mice receiving ID93/GLA-SE were not significantly different from RHZ treatment alone until week 12 (Figure 2). However, we do observe CD154, IFN-γ, IL-2, and TNF expression from the RHZ+ID93/GLA-SE groups as early as seven weeks post infection. At later timepoints following infection (weeks 16 and 22), therapeutic immunization induced a significantly higher frequency of CD4^+^ T cells producing CD154, GM-CSF, IFN-γ, IL-2, and TNF-α at 16 and 22 weeks post infection compared to RHZ-treated mice with ex vivo ID93 antigen stimulation (Figure 2). Immunized mice also induced higher levels of CD4^+^ IL-17A^+^ T cells by our final time point of 22 weeks post infection (Figure 2), similar to findings in human clinical studies [25]. This delay in antigen-specific responses suggest that the three-immunization regimen is critical to mounting this strong TH1 phenotype in the therapeutic schedule used here. It is evident that immunization with ID93/GLA-SE significantly induced changes in the magnitude and composition of CD4^+^ cytokine producing T cells compared to RHZ drug treatment alone. Additionally, these data show that ID93-specific TH1 responses are weak without immunization (RHZ group only).

### 3.2. Therapeutic Treatment with RHZ+ID93/GLA-SE Results in Significantly Reduced Pathology and Bacterial Burden Compared to Drugs Alone

**Figure 3:**
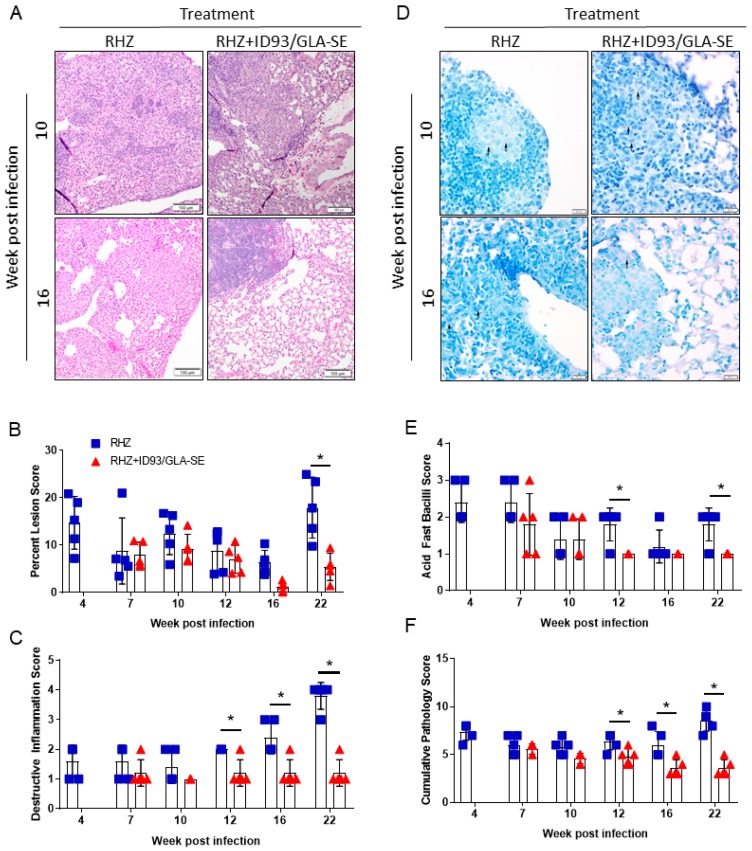
Decreased pathology with combined antibiotic and immunotherapy. (**A**) Representative H and E histological images from RHZ treated and RHZ+ID93/GLA-SE treated cohorts at 10 (upper) and 16 (lower) weeks post *Mtb* challenge. (**B**) Percent lesion area (mean ± SEM). (**C**) Destructive Inflammation score. (**D**) Representative acid-fast strained histological images from RHZ treated and RHZ+ID93/GLA-SE treated cohorts at 10 (upper) and 16 (lower) weeks post *Mtb* challenge. Acid-fast bacilli (AFB) are denoted by arrows. (**E**) AFB score and (**F**) cumulative pathology score of lung histology over time. Histological results are representative of two independent experiments. All data were evaluated using two-way ANOVA and Sidak’s correction for multiple comparison. The asterisk denotes significance where *p* < 0.05.

Although the mouse model of *Mtb* infection is often criticized for being dissimilar and too divergent from human pathology, recent reviews of inbred and Diversity Outbred mouse models highlight similarities, namely the necrotizing response to *Mtb* and granuloma formation [26,27]. Additionally, we have previously demonstrated significant changes in SWR/J mouse lung pathology and architecture after immunotherapy [17] and consider that this is a critical consideration for the evaluation of host-directed therapies against *Mtb.* Therefore, we next used histological analysis of the lung to determine the influence of immunotherapy combined with drug treatment on immunopathology following infection with *Mtb* H37Rv. The area of the lung encompassing lesions 22 weeks following *Mtb* infection was significantly decreased in mice receiving immunotherapy (ID93/GLA-SE) with drugs (RHZ) compared to those receiving drugs alone (Figure 3A,B). Furthermore, destructive inflammation, defined as cellular exodus or expansion from a well-defined granuloma into adjacent tissues, alveolar walls, and airways, is significantly lower in the group receiving therapeutic immunizations from week 12 through 22 post *Mtb* challenge (Figure 3C). We noted significantly more acid-fast bacilli in mice receiving antibiotics alone over time (Figure 3D,E). As the cumulative pathology score suggests (Figure 3F), therapeutic immunization in combination with antibiotics provides significant protection from damaging pathology beginning at 12 weeks and continuing through our assessment at 22 weeks post infection.

Representative histological images from each time point assessed can be found in Figure S2. Interestingly, despite these pathological indicators of reduced local lung inflammation and damage during chronic infection we found no significant differences in C-reactive protein (CRP) levels in the serum between cohorts of mice receiving RHZ alone, or in combination with immunization (Figure S2, Supplementary Materials). Although plasma CRP has been demonstrated to be elevated in active cases versus household contacts [28], and is significantly higher in culture positive versus culture negative TB cases [29], levels of CRP do steadily decrease after intensive treatment in human trials [29,30,31]. Since our preclinical model includes eight weeks of RHZ antibiotics we are likely not able to resolve differences in CRP levels immunotherapy may induce. These data may suggest therapeutic immunizations have a local effect on controlling bacterial burden, but little to no complementary influence on systemic inflammation above that induced by antibiotic alone in the infected host during the therapeutic window investigated. Importantly, therapeutic immunizations provide a significant protective advantage from lung pathology over antibiotic treatment alone.

**Figure 4:**
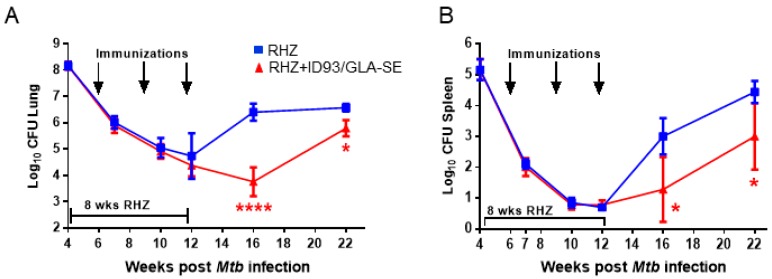
Additive control of bacterial burden with therapeutic ID93/GLA-SE vaccination over time. Log_10_ bacterial burden in the (**A**) lung and (**B**) spleen over time. Bacterial burden is represented as Log_10_ colony forming units (CFU) following RHZ treatment (blue, squares) or RHZ+ID93/GLA-SE (red, triangles), and was assessed by two-way ANOVA with Sidak’s multiple comparisons test. **** *p* < 0.0001, * *p* < 0.05. Seven mice/group/timepoint were included, and data are representative of two independent experiments.

As expected, periods of reduced bacterial burden (as determined by CFU counts) in those cohorts receiving immunizations coincided with reduced immunopathology. Specifically, we detected significantly lower CFU in the lungs and spleens of ID93/GLA-SE immunized mice at 16 and 22 weeks post infection (Figure 4A,B). Furthermore, we found that immunization with ID93/GLA-SE was able to provide a further reduction in organ CFU over that of antibiotics alone from week 12 to week 22 post infection, suggesting these two treatments (drugs and ID93/GLA-SE) have an additive effect for controlling pulmonary and disseminated *Mtb* (Figure 4). Immunotherapy and drug treatment reduced bacterial burden by 2.64 and 0.78 Log_10_ CFU in the lung at 16 and 22 weeks post infection, respectively. Similarly we noted a 1.72 and 1.44 Log_10_ CFU reduction at 16 and 22 weeks post infection in the spleen, respectively, with a combination of drugs and immunotherapy (Figure 4). Although the reduction in bacterial burden at 16 and 22 weeks post infection is robust, there was a trending increase in CFU from week 16 to 22 suggesting that this bacterial control may be transient. Of interest, this correlates with a plateau of TH1 cytokines produced between weeks 16 and 22 from CD4^+^ T cells in the immunized group as seen by intracellular flow cytometry (Figure 2).

These data taken together indicate that a strong TH1 response is beneficial for therapeutic vaccines strategies, but is only partly able to control the bacterial burden over time and that a plateaued response may result in an accumulation of *Mtb* post treatment. Despite this trending increase in CFU from week 16 to week 22 post infection, we note that negative pathology scores remain significantly lower in the immunized group, suggesting that enumeration of bacterial load may not be the sole appropriate indicator of successful therapy and that other influences, such as granuloma containment, may be greatly influenced by immunization regimens. Indeed, this kind of local lung granuloma containment may directly reduce active disease spread.

### 3.3. Antibiotics and Immunotherapy Reduce the Overall Inflammatory Status of an *Mtb*-Challenged Host

**Figure 5:**
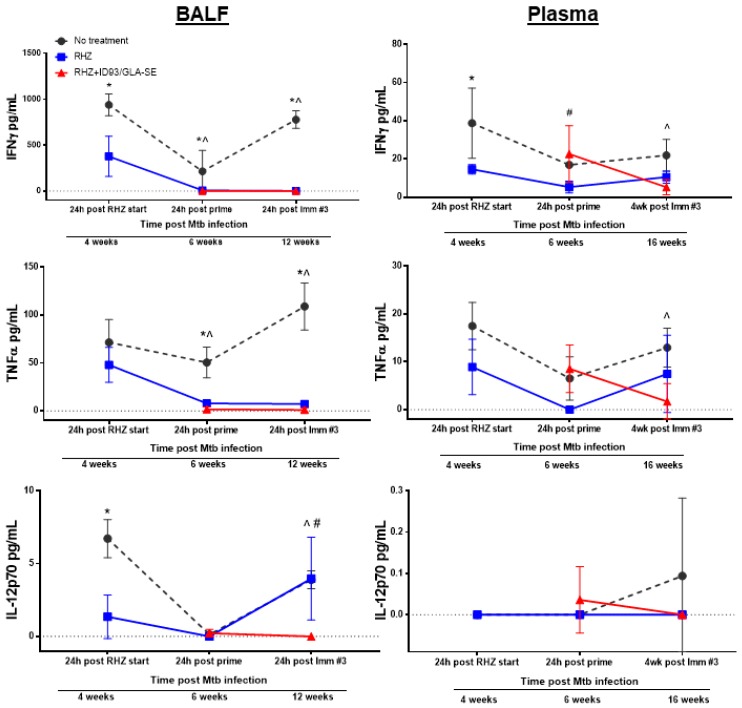

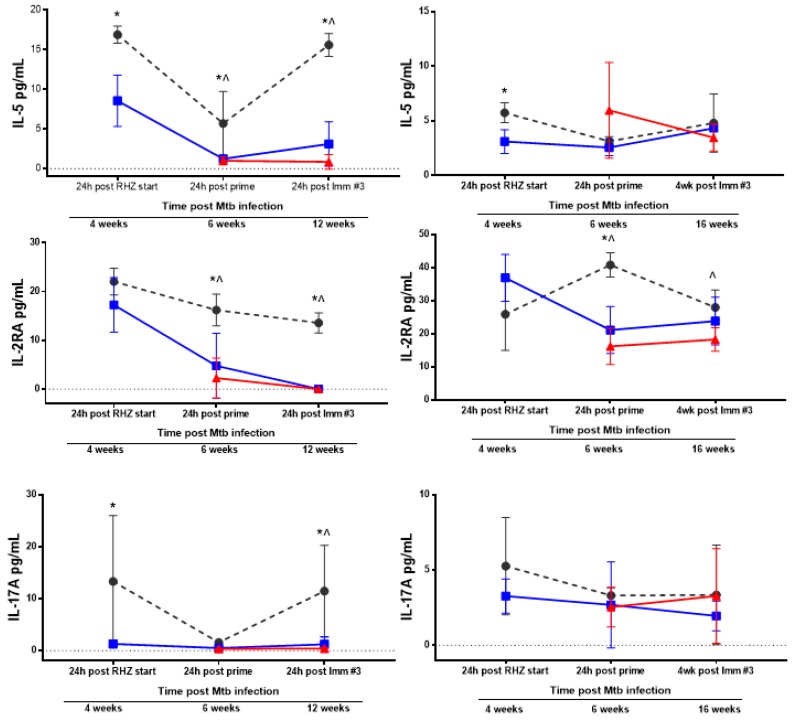
Therapeutic treatment against *Mtb* leads to reduced BALF and plasma inflammatory cytokines over time. Cytokine and chemokine analysis of BALF (**left**) and plasma (**right**) samples from cohorts of mice that were either untreated (grey, circles), RHZ-treated (blue, squares), or RHZ+ID93/GLA-SE-treated (red, triangles). The data represents the average value ± SD of four or five individual mice run in duplicate for each time point. Time points examined included 24 h post RHZ initiation, 24 h post prime and either 24 h or three weeks following the final immunization, corresponding to 4, 6, and 12 weeks (BALF) or 4, 6, and 16 weeks (plasma) post *Mtb* infection. Groups were compared using two-way ANOVA and Sidak’s multiple comparisons test. *p* < 0.05 difference between groups at a specific time point represented as: * no treatment versus RHZ; ^ no treatment versus RHZ+ID93/GLA-SE; # RHZ versus RHZ+ID93/GLA-SE.

Next, we sought to determine the non-specific inflammatory landscape within the lung milieu and circulation as an indicator of safety. Subsequently, we expanded this examination to include an untreated group to further scrutinize changes induced by antibiotics alone and the effects which may be attributed to ID93/GLA-SE immunotherapy. Analysis of 26 different analytes by Luminex, chosen for their documented roles in *Mtb* control in humans or mouse models, was performed at various timepoints following infection with *Mtb* H37Rv to correlate treatment related outcomes of different treatment regimens. Notably, we selected analytes that demonstrate elevated expression in TB patients compared to household contacts or controls, classify disease severity or susceptibility in preclinical models of infection, or are elevated in cases of recurrence, including: CXCL5 (ENA-78) [32,33], GM-CSF [34,35], IL-1β [28,36], IL-2 [32,35], IL-2RA [37,38], IL-5 [39], IL-6 [28,40,41], IL-9, IL-12p70 [42], IL-13 [35], IL-15/15R [43], IP-10 (CXCL-10) [35,40,43], MCP-1 [43], MIP1-β [35], and TNFα [36,37,40]. We also assessed IL-7RA [44], Leptin [45], and IL-17a [46], which have lower expression during TB infection and correlate with worse disease. BALF and plasma samples were acquired from untreated, RHZ only, or RHZ+ID93/GLA-SE cohorts over time (as described). RHZ and RHZ+ID93/GLA-SE treatments generally lowered levels of inflammatory cytokines over time compared to no treatment groups, with few exceptions (Figure 5). Specifically, we noted elevated levels of IL-5 in BALF samples from untreated animals compared to either treatment (Figure 5). This elevation in IL-5 may be indicative of a skewed immune response that is more TH2-like, which can be associated with poorer outcomes in TB infection [16]. Most analytes, including IFN-γ, TNFα, IL-2RA, and IL-17A, were decreased in BALF from RHZ and RHZ+ID93/GLA-SE treatment groups compared to no treatment over time, whereas IL-12p70 was only significantly lower in BALF from the RHZ+ID93/GLA-SE group, but not different in plasma samples (Figure 5). Interestingly, IL-6, IP-10, CXCL5, and MIP1-β levels were higher 12 weeks post infection in groups receiving no treatment, whereas all other time points were similar between each of the three groups (data not shown). Along with histology, these analyses suggest that antibiotic treatment and a combination of antibiotics with immunotherapy reduce the non-specific inflammatory state within the lung while still allowing specific proinflammatory responses (Figure 2) to occur. This may suggest that excessive inflammation and TH2 biased immune responses are detrimental to the host and indeed a controlled and localized TH1 response is favorable for *Mtb* control.

### 3.4. The Percentage of Regulatory T Cells in the Lung Are Not Increased Due to Immunotherapy in the Context of Drug Treatment

**Figure 6:**
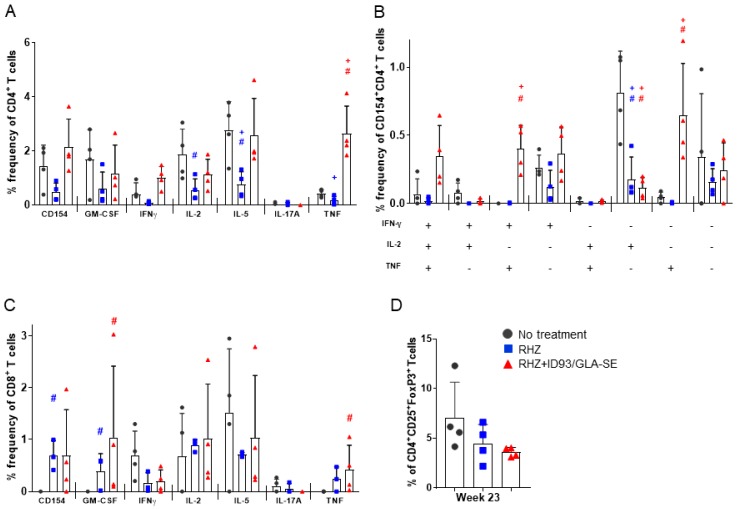
Increased ID93-specific cytokine responses from CD4^+^ and CD8^+^ T cells in the lung following immunotherapy. Pulmonary T cell responses assessed 23 weeks after *Mtb* infection. (**A**) The percent of single-cytokine producing CD4^+^ T cells; (**B**) the percent of polyfunctional CD4+ T cells producing one or more cytokines; and (**C**) the percent of single-cytokine producing CD8^+^ T cells, examined by intracellular flow cytometry after ex vivo stimulation with ID93 antigen. Data are representative of two individual experiments. Groups were compared by Student’s *t*-test and the Wilcoxon rank test, and significance is denoted by # and +, respectively. (**D**) The percent of regulatory T cells, CD4^+^FoxP3^+^CD25^+^ 23 weeks post infection from untreated (dark grey, circles), RHZ (blue, squares), or RHZ+ID93/GLA-SE (red, triangles) treated mice. Comparisons between groups performed using one-way ANOVA with Sidak’s multiple comparison test were all found to be not significant (n.s.).

Intracellular cytokine flow cytometry at 23 weeks post infection confirmed the proinflammatory ID93-antigen specific responses seen previously (Figure 2). Whereas animals treated with RHZ alone demonstrated very minimal TH1 responses, untreated cohorts demonstrated nearly equivalent magnitude of cytokine induction post stimulation as RHZ+ID93/GLA-SE-treated cohorts (Figure 6A), with the exception of TNF, which was significantly higher in immunized animals. In addition, RHZ-only-treated animals demonstrated significantly less production of IL-5 and IL-2 by CD4^+^ T cells compared to untreated animals. We also noted a trend toward higher induction of polyfunctional IFN-γ^+^IL-2^+^TNF^+^ CD4^+^ T cells, and a significantly higher percentage of IFN-γ^+^TNF^+^ CD4^+^ T cells over that of untreated animals in the RHZ+ID93/GLA-SE group. RHZ treated cohorts demonstrated a significantly muted IL-2 antigen specific response (Figure 6B). These data demonstrate that the magnitude and composition (cytokines produced) of the TH1 response is directly influenced by immunotherapy with ID93/GLA-SE. The evaluation of CD8^+^ T cells after ID93 stimulation revealed a significantly greater percentage of cells inducing TNF in the immunized groups compared to untreated, but no overall trend of proinflammatory responses (Figure 6C). Additionally, we noted little to no evidence of antigen-specific polyfunctional CD8^+^ T cell responses in any of the cohorts (data not shown). The intracellular cytokine responses induced at 23 weeks were similar to those evaluated at 17 weeks post infection (data not shown). Despite the modulated nature of proinflammtory CD4^+^ T cell responses (Figure 2), changes in cytokines noted in BALF or plasma (Figure 5) and histological dissimilarity (Figure 3) over time we noted no significant difference in the percentage of CD4^+^ regulatory T cells in lung homogenate at 23 weeks post infection between groups (Figure 6D). The comparable levels of regulatory T cells in the lung at 23 weeks post infection, suggests at this timepoint the plateau of proinflammatory responses is not due to this specific cellular intervention and might instead elude to a peak response reached without further stimulation or modulation by some other factor.

### 3.5. Immunization Provides a Significant Additive Survival Advantage over That of Antibiotics Alone

**Figure 7:**
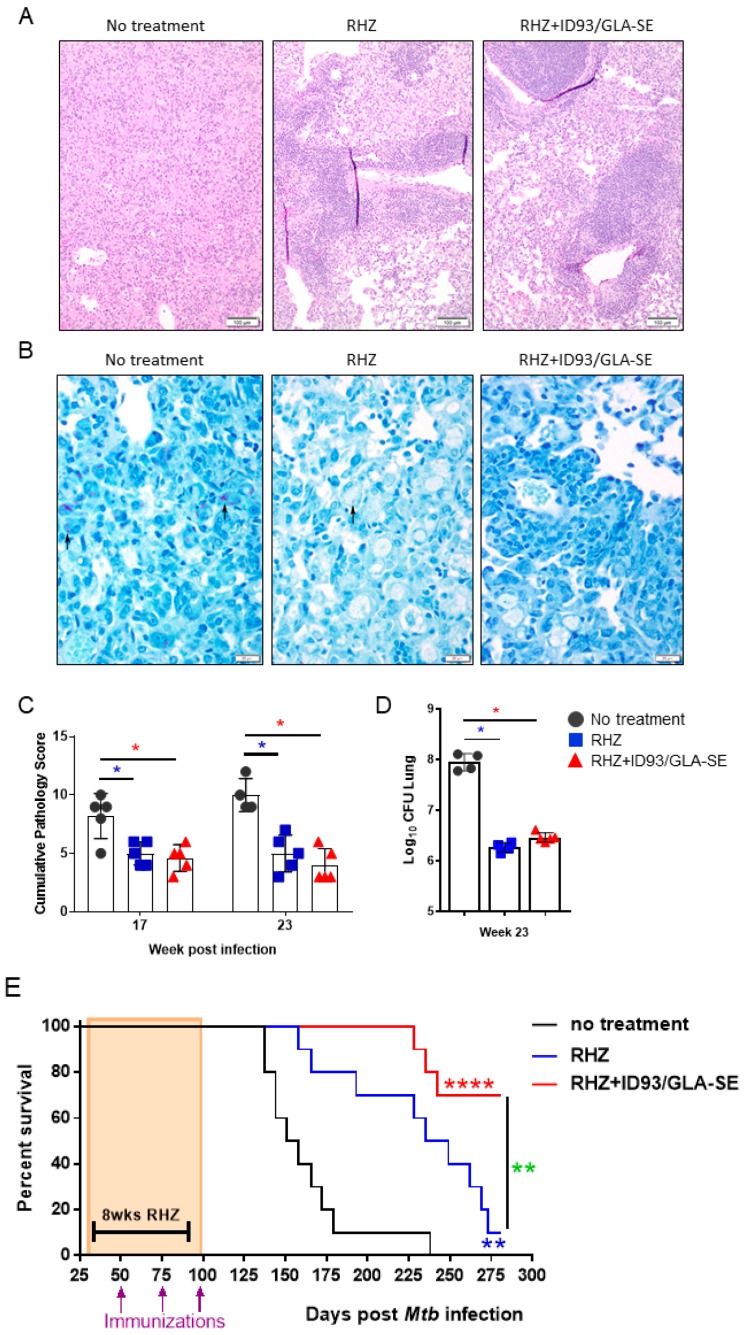
Improved survival with RHZ drug treatment plus immunotherapy. (**A**) Representative H and E and (**B**) AFB histological images from mice that received no treatment, or RHZ or RHZ+ID93/GLA-SE treatment 23 weeks post *Mtb* challenge. AFB denoted by arrows. (**C**) The cumulative score of lung pathology over time. Histological analyses are representative of two independent experiments. All data were evaluated using two-way ANOVA and Sidak’s correction for multiple comparison. The asterisk denotes significance where *p* < 0.05. (**D**) Bacterial burden in the lungs 23 weeks post infection from untreated (dark grey, circles), RHZ (blue, squares) or RHZ+ID93/GLA-SE (red, triangles) treated mice. Comparisons between groups performed using one-way ANOVA with Sidak’s multiple comparison test. *p* < 0.05 denoted by an asterisk. (**E**) The percent survival over time of cohorts of 10 mice/group that received no treatment (dark grey), RHZ (blue), or RHZ+ID93/GLA-SE (red) treatment. Groups were compared using Log-rank (Mantel Cox) test, and asterisks represent a significant difference between groups: blue = no treatment v RHZ, red = no treatment v RHZ+ID93/GLA-SE and green = RHZ v RHZ+ID93/GLA-SE. ** *p* < 0.01 and **** *p* < 0.0001.

Both RHZ- and RHZ+ID93/GLA-SE-treated cohorts demonstrate a nearly identical and significant reduction in lung bacterial burden at 23 weeks post infection compared to untreated mice (Figure 7D). This similar level of bacterial burden is unsurprising based on the steady increase in CFU from weeks 16 to 22 previously seen (Figure 4). In addition to the comparable levels of CFU at 23 weeks post infection we see significantly reduced lung pathology in both RHZ and RHZ+ID93/GLA-SE treatment compared to untreated groups (Figure 7A–C) suggesting *Mtb* may indeed be better controlled in the groups receiving therapeutic immunizations. This is supported by an analysis of survival over time between groups. Mice receiving antibiotics alone survive significantly better than untreated mice over time and succumb to infection around 280 days post infection, whereas in cohorts of mice receiving antibiotics and therapeutic immunizations with ID93/GLA-SE survival is significantly improved over antibiotics alone, and as of 300 days post infection, over 70% of mice had yet to succumb to infection (Figure 7E). Taken together, these data advocate for an added immunologic, pathologic, and survival advantage of therapeutic immunizations over that of antibiotic treatment alone.

## 4. Discussion

We aim to design therapeutic vaccines that will prevent recurrence in infected individuals and prevent active disease states in order to reduce transmission and hopefully halt/slow the rapid spread of DS and DR strains of *Mtb* globally. Here we demonstrate that immunotherapy with ID93/GLA-SE provides an additive bacterial burden control over antibiotic treatment alone, which corresponds with periods of robust CD4^+^ TH1 T cell responses in the lung. Importantly we noted significantly improved lung pathology in groups that received ID93/GLA-SE in the context of drug treatment, which may be directly responsible for the enhanced survival seen in this highly-susceptible SWR/J mouse model. The gradual increase of ID93-specific TH1 responses within the lung seems desirable in the context of *Mtb* infection, rather than an uncontrolled chronic proinflammatory profile similar to that observed in untreated mice. This may help reduce the undesirable influx of too many proinflammatory, tissue damaging cells (such as neutrophils), but allow enough influx to promote a lymphocytic-enriched granuloma. The nature of the granuloma induced with antibiotics and immunotherapy may reduce the intracellular niches for *Mtb* within the lungs and provide more elegant control of *Mtb*, and warrants further investigation. These data expand upon our previous understanding of the influence immunotherapy has on the systemic and local inflammatory response, and suggests a significant role for maintaining pulmonary architecture in the improved survival with immunotherapy.

The seemingly transient control of *Mtb* demonstrated here suggests that there are aspects of therapeutic vaccination that mediate the reduced CFU burden, but may not provide lasting protection. Future investigations should examine the role of the vaccination schedule, specifically the intervals between immunizations, to determine whether this would allow for a more rested T cell phenotype to emerge. Similarly, delaying therapeutic immunizations until the end of treatment (EOT) may reduce the overall antigen burden stemming from *Mtb* infection, which could mask the generation of protective cellular immunity induced by immunotherapy, leading to dampened bacterial control over time. We suspect that antigen load at the time of immunization will directly influence subsequently derived T cell phenotypes. Adding a bolus of antigen in a chronic inflammatory setting, in the form of therapeutic vaccination for example, may drive terminal T cell differentiation instead of the desired memory subsets, as demonstrated by Andersen et al. in their therapeutic study describing the detrimental effects of high antigen dose in a post-*Mtb* exposure setting [47]. Indeed in our experimental design we chose to abstain from including an immunization only arm (in the absence of RHZ drug treatment) since, in preclinical models, this regimen can induce significant morbidity presumably by exacerbating the proinflammatory environment within the lung. Furthermore, the chronic nature of the infection can obscure changes in the immune profile induced by therapeutic immunizations (unpublished results). This, along with anecdotal clinical trial evidence (including a phase 2 clinical trial for *Mtb* antigen vaccine candidate M72 and AS01E adjuvant from GlaxoSmithKline (trial identifier: NCT01424501), was terminated early due to adverse immune events (injection site reactions) in the groups of individuals who were still taking antibiotic treatment for TB at the time of vaccination, while no adverse events were noted in groups that had completed antibiotic treatment [4]) make this therapeutic immunization strategy a less viable translational option. Connecting antigen burden during antibiotic treatment and safe windows of therapeutic immunizations will be important for moving therapeutic vaccine candidates forward and, importantly, these dynamics may vary between candidates that use different *Mtb* antigens.

Our demonstration of a plateaued TH1 response and bacterial burden increasing from week 16 to week 22 may point to a defect in memory formation, resulting from phenotypic skewing towards exhaustion and T cell anergy [48]. It will be important to further investigate these findings in the context of recent correlates of risk (COR) signatures discovered empirically in human clinical trial samples [49,50] and associated with similar risk signatures displayed in a nonhuman primate model of tuberculosis [51], as well as cutting-edge immune signatures of latent TB uncovered via population transcriptome analysis [52]. Indeed, the bacterial spatial control and maintenance of lung architecture with immunotherapy described here for RHZ+ID93/GLA-SE should continue to be evaluated for active versus latent TB disease markers. Furthermore, several analytes associated with baseline disease severity, culture conversion, and treatment response in human clinical trials should be assessed in combination with those evaluated here [41]. In addition, adapting drug treatment to closely mimic new regimens being used in clinical trials, such as the use of bedaquiline [53], would be worthwhile.

## 5. Conclusions

Here, we have demonstrated that therapeutic immunizations with anti-*Mtb* vaccine candidate ID93/GLA-SE in combination with RHZ drug treatment induces (1) robust antigen specific TH1 responses that correlate with periods of enhanced bacterial control, (2) better survival over time and (3) present histological evidence that suggests vaccine-mediated protection from damaging lung pathology compared to drug treatment alone. We believe these data may help shape appropriate therapeutic immunization strategies for DS and DR *Mtb*. It is evident in the historical shortcoming of TB vaccine candidates, that the future success of therapeutic immunization regimens are dependent on understanding the inflammatory state of the host at the time of immunization and subsequent immune phenotypes that are generated. This study also underscores that immunotherapy results in a better immune profile and the control of physiopathological processes accompanying disease development in TB, which are implied for clinical recovery. Understanding the nuanced contributions to immune system regulation has the potential to dramatically impact the global health burden of TB disease.

## Supplementary Materials

The following are available online at http://www.mdpi.com/2076-393X/6/2/30/s1, Figure S1. Gating strategy for flow cytometry analysis; Figure S2. Representative histology from each time point assessed.
