# Supplementary material for: Enhanced Anti-Mycobacterium tuberculosis Immunity over Time with Combined Drug and Immunotherapy Treatment
Source: Vaccines (Basel). 2018 May 24;6(2):30. doi: 10.3390/vaccines6020030 (PMC6027321; doi:10.3390/vaccines6020030)
Supplement: Supplementary file 1 [file vaccines-06-00030-s001.pdf]

Cytokine stimulation

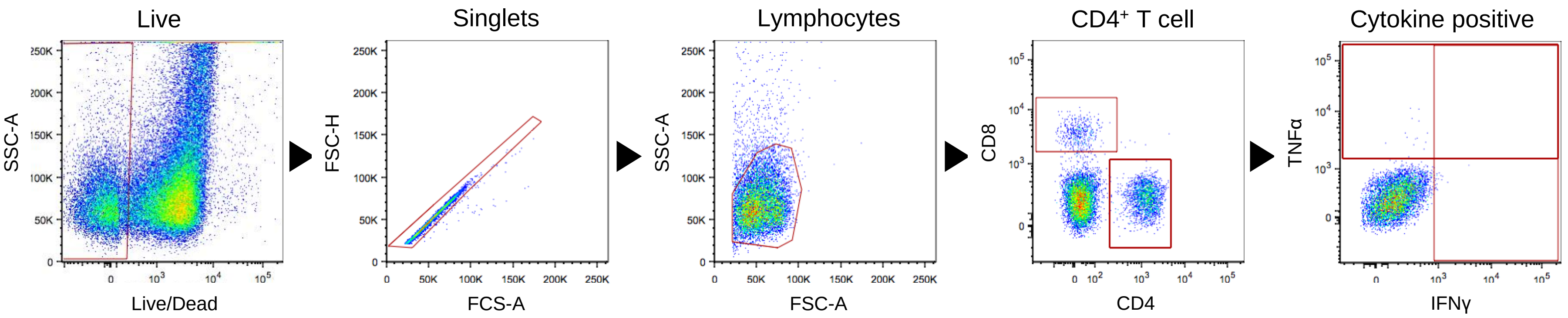

TREGS

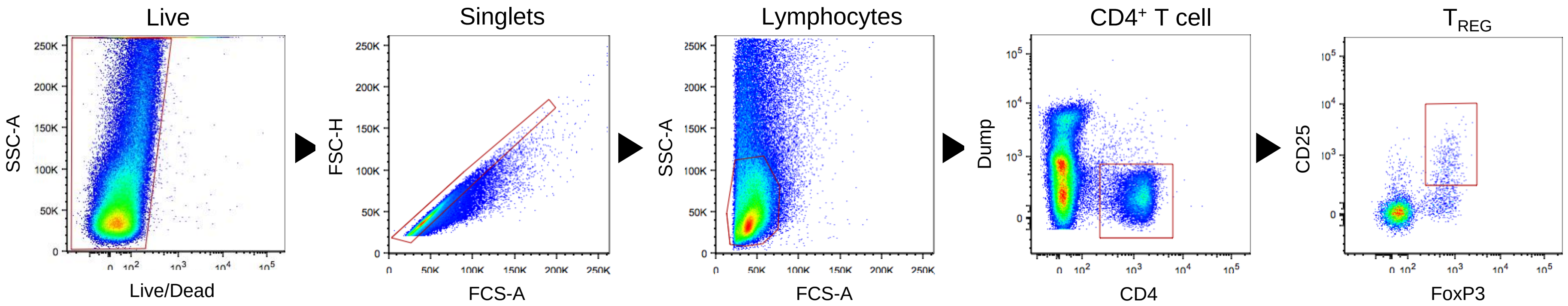

Supplementary Figure 1: Gating strategy for intracellular cytokine staining and regulatory T cell evaluation.

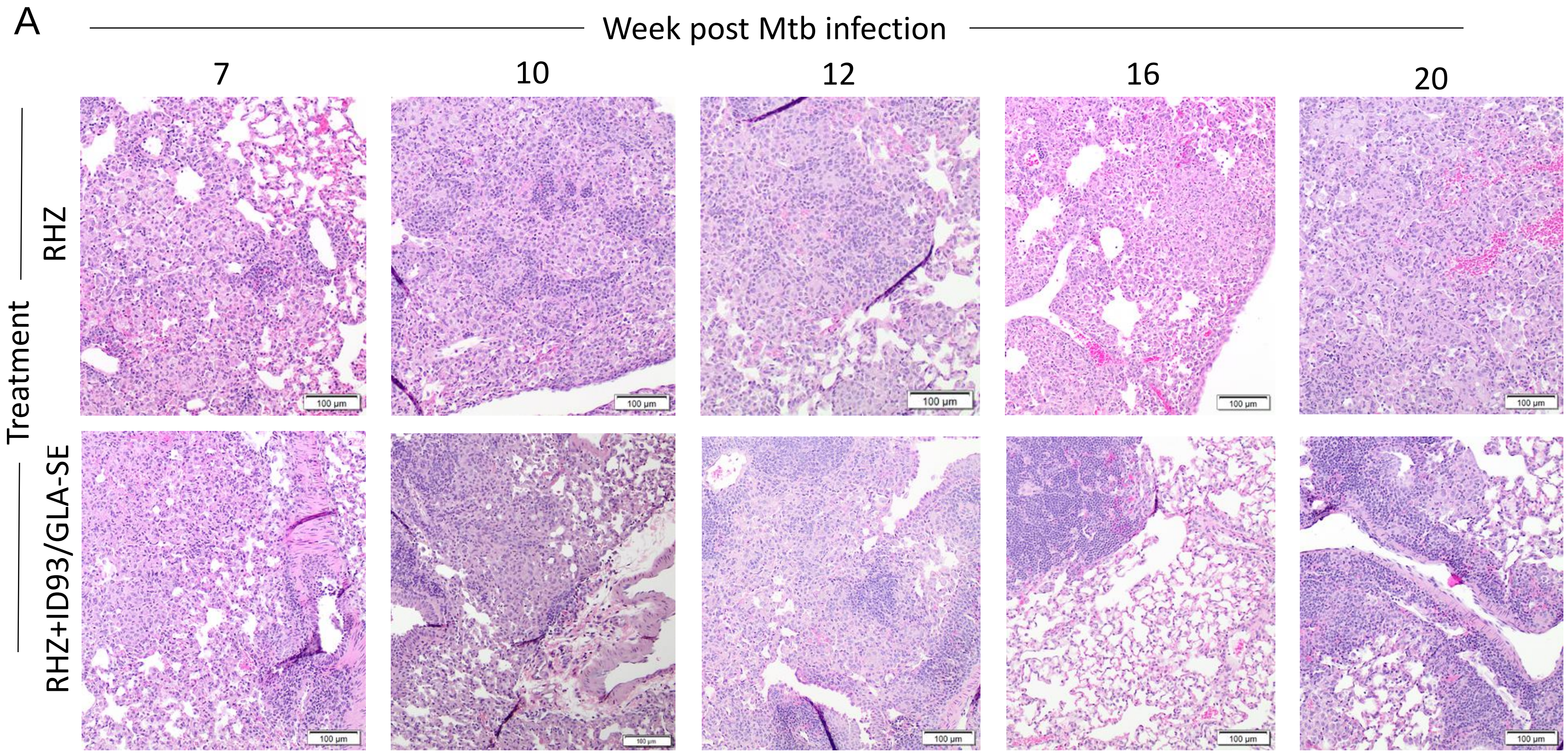

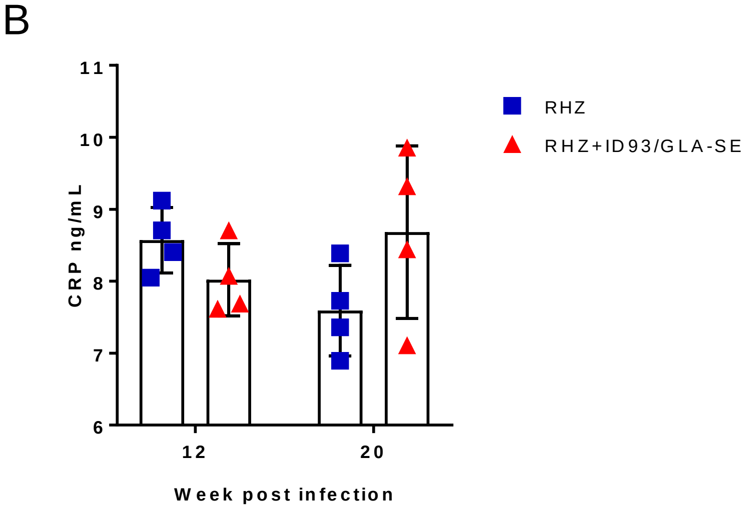

Supplementary Figure 2: **Representative histology slides from each treatment group**: A) RHZ alone (top) or RHZ+ID93/GLA-SE (bottom) over time. Data are representative of two independent experiments. B) C-reactive protein (CRP) levels detected in the plasma of RHZ or RHZ+ID93/GLA-SE treated mice at 12 or 20 weeks post infection. Data were evaluated using a 2-way ANOVA and Sidaks correction for multiple comparison, and considered not significant based on p <0.05. Four mice/group/timepoint were included and were representative of two independent experiments.
